# EEG Spectral Exponents and Visual Chirp Responses Mirror Anti‐Seizure Medication Load in Refractory Focal Epilepsy

**DOI:** 10.1002/acn3.70045

**Published:** 2025-04-21

**Authors:** Silvano R. Gefferie, Arthur R. van Nieuw Amerongen, Gerhard H. Visser, Maeike Zijlmans, Else A. Tolner, Mark van de Ruit, Arn M. J. M. van den Maagdenberg, Roland D. Thijs

**Affiliations:** ^1^ Stichting Epilepsie Instellingen Nederland (SEIN) Heemstede the Netherlands; ^2^ Department of Neurology Leiden University Medical Centre Leiden the Netherlands; ^3^ Department of Neurology University Medical Center Utrecht Utrecht the Netherlands; ^4^ Department of Human Genetics Leiden University Medical Centre Leiden the Netherlands; ^5^ Department of Biomechanical Engineering Delft University of Technology Delft the Netherlands; ^6^ Department of Clinical & Experimental Epilepsy UCL Queen Square Institute of Neurology London UK

**Keywords:** anti‐seizure medications, electroencephalography, photic stimulation, resting state EEG, spectral analysis

## Abstract

**Objective:**

Quantitative markers of cortical excitability may help identify responders to anti‐seizure medications (ASMs). We studied the relationship between ASM load and two electroencephalography (EEG) markers of cortical excitability in people with refractory epilepsy.

**Methods:**

We included individuals with refractory focal epilepsy undergoing presurgical evaluation, involving ASM tapering and sleep deprivation. We obtained daily resting state EEG and EEG responses to visual stimulation at linearly increasing flash frequency (10–40 Hz chirp). We extracted the aperiodic exponent from resting state EEG power spectra and analysed chirp response at driving and second‐order harmonic frequencies. We modelled ASM load, which we related to the EEG markers using linear mixed‐effects regression.

**Results:**

Forty‐eight subjects (median age 34 years, age range 16–62 years, 19 females) participated. The spectral exponent became less negative with ASM load reduction (*p* = 0.02), mainly attributable to reduced low‐frequency power. Lowering ASM load increased the harmonic response to chirp stimulation (*p* = 0.004), also after accounting for sleep deprivation (*p* = 0.02), but did not affect the driving response. ASM tapering specifically increased harmonic responses to high stimulation frequencies (27–40 Hz, *p* = 0.01).

**Interpretation:**

Resting state EEG spectral exponents and visual chirp responses reflect ASM load in refractory epilepsy. Low‐frequency spectral changes in resting state EEG may only mirror ASM‐induced spectral slowing. Visual chirp stimulation reveals enhanced harmonic EEG responses during low ASM loads, likely due to both increased high gamma activity and increased response to visual perturbations. Implementation of the markers would need normative values to reduce the delay to individually optimised treatment regimens.

## 1 Introduction

Clinical management of epilepsy is fraught with a lack of biomarkers to track anti‐seizure medication (ASM) efficacy [1], which is currently evaluated via a lengthy trial‐and‐error process. ASMs are typically considered to reduce seizure burden by restoring the pathologically enhanced cortical excitability [2, 3, 4] that represents a characteristic trait of the epileptic brain [5]. Hence, ASM‐induced normalisation of the level of brain excitability may predict treatment success. Biomarkers of cortical excitability could thereby help assess ASM efficacy, but non‐invasive and easily accessible excitability measures sensitive to ASM effects remain to be identified.

Cortical excitability can be indexed either by deriving excitability measures from spontaneous brain activity or by probing the brain's response to perturbations. Concerning excitability measures from spontaneous activity, the incidence of interictal electroencephalographic (EEG) events including epileptiform spikes and pathological high‐frequency oscillations (HFOs) has been related to enhanced neuronal excitability [6, 7]. Whereas effects of ASMs on spike rates appear variable [8, 9, 10], HFO rates have been demonstrated to increase with reduced ASM intake [11]. Unfortunately, monitoring HFOs demands high sampling frequencies and, though efforts have been made towards algorithmic approaches [12], elaborate visual analysis of the EEG is currently still required. Spectral EEG analysis potentially offers an easily accessible approach for assessing ASM effects [13]. Measures of the neuronal network excitation‐to‐inhibition (E/I) ratio, which correlate with cortical excitability [14], appear embedded in the power spectral density (PSD) of spontaneous cortical signals [15]. A less negative ‘aperiodic’ spectral exponent (or: a flatter slope) of the 1/f approximation of the PSD was indicated to reflect an increased E/I ratio, both in preclinical [15] and human [16] data. Compared to healthy controls, the interictal spectral exponent was found more negative in the context of epileptic encephalopathy [17] but less negative in people with generalised epilepsy [18]. Smaller exponents were found to be specifically associated with cases of sleep‐related hypermotor epilepsy, when compared to NREM parasomnia [19]. The spectral exponent appears to become more negative during epileptiform activity and a stronger circadian modulation was suggested to be predictive of better ASM efficacy [20].

With respect to using perturbations, transcranial magnetic stimulation has been shown to enable robust assessment of ASM‐driven cortical excitability changes [3, 21]. Transcranial magnetic stimulation, however, requires expensive equipment and, especially when combined with EEG [22, 23], complex experimental procedures. Visual stimulation represents an attractive alternative, easily accessible means of indexing cortical excitability based on visual responsivity characteristics, especially in photosensitive epilepsy [24, 25, 26, 27, 28, 29]. Visual ‘chirp’ stimulation represents a generalised form of steady‐state photic stimulation [30], as it features a non‐zero rate of change of stimulation frequency rather than a fixed stimulation frequency during stimulus trains. The use of a wide range of visual stimulation frequencies allows for comprehensive and rapid evaluation of frequency‐dependent EEG responses, including resonance phenomena [31]. Compared to current approaches that use intermittent photic stimulation to probe cortical excitability, visual chirp stimulation does not rely on photoparoxysmal responses, which occur in only a subset of people with epilepsy [32]. In migraine, EEG responses to visual chirp stimulation were more pronounced in cases compared to controls [33] and during pre‐ictal compared to interictal episodes [34].

We here aimed to determine whether the spectral exponent of resting state EEG and the EEG response to visual chirp stimulation can track ASM‐dependent dynamics of cortical excitability. We evaluated these EEG markers in people with refractory epilepsy undergoing presurgical evaluation.

## 2 Materials and Methods

### 2.1 Participants

We recruited individuals, aged 16 years or older, admitted to the Epilepsy Monitoring Unit of Stichting Epilepsie Instellingen Nederland (SEIN) for presurgical evaluation between June 2022 and October 2023. All participants were diagnosed with or suspected of having refractory focal epilepsy at study entry. Withdrawal of ASMs, oftentimes starting a few days prior to admission, and partial nocturnal sleep deprivation (pNSD) were among the seizure provocative procedures, tailored to individual clinical plans. For each participant, we extracted clinical characteristics from electronic medical records and documented the ASM regimen at home and during admission, alongside the administration of rescue medication, seizure occurrence and pNSD for each in‐patient day. The regional ethical board (NedMec, University Medical Center Utrecht) evaluated the study protocol and concluded that the Dutch Medical Research Involving Human Subjects Act did not apply, such that formal approval was not required. We obtained written informed consent from all individuals before inclusion.

### 2.2 Data Acquisition

EEG was recorded using SystemPlus Evolution software (Micromed S.p.A, Treviso, Italy), with 19 electrodes at 10–20 positions and two electrodes, F9 and F10, at 10–10 positions. Based on the individual clinical question, supplementary electrodes were applied according to the 10–10 system. Reference and ground electrodes were located at CP1 and CP2 locations. Initial impedances were 3 kΩ or lower and regularly corrected. The EEG was sampled at 256 Hz.

We performed daily measurements of resting state EEG activity and EEG responses to visual chirp stimulation at around 2 p.m. The measurement time was delayed in the case of a recent focal (< 1 h) or convulsive (< 2 h) seizure, or advanced if the participant was to be discharged by noon. During the measurements, the participants were seated in bed with their eyes closed and muscles relaxed. We used Psytoolkit [35, 36] to instruct the participants and to regularly play voice notes aimed at keeping them awake. The resting state EEG measurement consisted of two 3.5‐min epochs, separated by a 15‐s break. For subsequent visual chirp stimulation, we used binocular yellow‐light (wavelength 590 nm) LED goggles (Micromed S.p.A., Treviso, Italy) with a luminous intensity of 223.2 cd per eye. The goggles were controlled by custom‐written scripts [34] in MATLAB R2021a (The Mathworks Inc., Natick, MA, USA). The visual chirp stimulation, following a previous study [33], involved 12 trials that were interspaced by intervals of randomised duration between 10 and 15 s. Each trial consisted of flashes of linearly increasing frequency. Specifically, four flashes were presented at each frequency between 10 and 40 Hz (1‐Hz increments), totalling 124 flashes. The duration of each trial was 5.7 s. Digital markers that indicated the beginning of each chirp trial in the EEG recordings were stored for data pre‐processing.

### 2.3 Data Analysis

We used custom‐written scripts, and the EEGLAB [37] and FieldTrip [38] toolboxes, in MATLAB R2023b (The MathWorks Inc., Natick, MA, USA) to pre‐process and analyse the EEG recordings.

**FIGURE 1:**
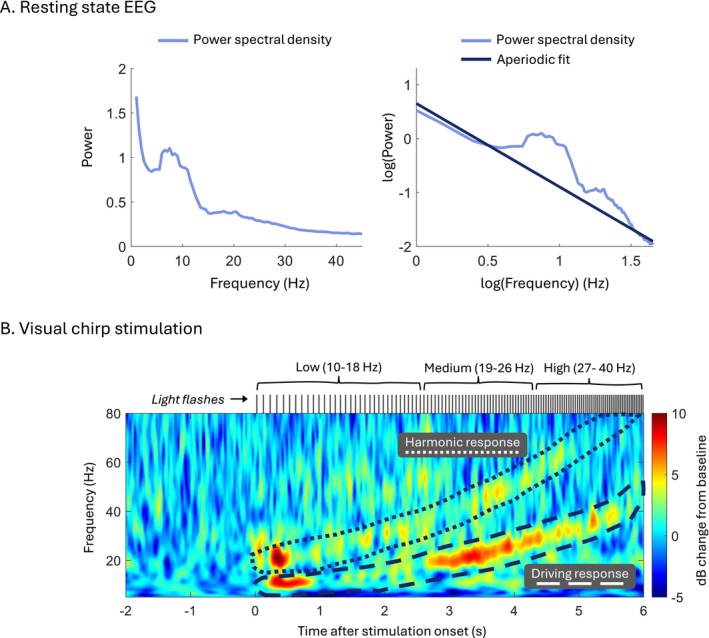
Extraction of quantitative markers from resting state electroencephalographic (EEG) data and EEG responses to visual chirp stimulation. (A) Power spectral density of an example central channel's resting state EEG (left), and the fit of the 1/f (aperiodic) component, demonstrated in the log–log space (right). The slope of the fitted aperiodic component in the log–log space equals the spectral exponent fit in the original space. (B) Time‐frequency representation of a representative example EEG response to visual chirp stimulation, averaged across 12 chirp trials. Each trial, lasting 5.7 s, presents flashes of linearly increasing frequency, with four flashes at each integer frequency between 10 and 40 Hz (upper black vertical lines). The average chirp response is baseline‐corrected using the average 1.5‐s interval preceding the stimulus onset from the participant's first available recording session. The responses at stimulation frequencies (dashed outline) and doubles of the stimulation frequencies (dotted outline) are averaged to arrive at a single driving and second‐order harmonic response, respectively. The driving and harmonic responses are further decomposed into a response at low, medium, and high stimulation frequencies.

For each resting state EEG recording, we merged the two 3.5‐min epochs. We detected bad channels with the clean_artifacts.m function from EEGLAB, which checks channels for low correlation to other channels (< 0.9), high line noise‐to‐signal ratio (> 4) and long duration of a flat line signal (> 1 s). We removed a channel if it contained interictal spikes or muscular, ocular, or cardiac activity for at least 50% of the recording. Frontopolar channels (Fp1 and Fp2) were always removed due to excessive ocular artefacts. Removed midline channels (Pz, Cz and Fz) were interpolated. The remaining channels were re‐referenced to a point at infinity [39] and simultaneously visually inspected for any residual artefacts, which we rejected. The resulting data fragments were recombined by autoregressive modelling [40] using a 100‐millisecond two‐sided margin. After applying a notch filter at 50 Hz, we estimated the PSD (Welch's method, 2‐s Hamming windows, 50% window overlap) for each channel for the first 120 s of artefact‐free data. We applied the Fitting Oscillations and One‐Over‐F (FOOOF) algorithm [41] in MATLAB R2023b (The MathWorks Inc., Natick, MA, USA) to estimate the aperiodic component of the channels' PSDs and extract their exponents (Figure 1). We used a fitting range of 1–45 Hz and a spectral peak width limit between 1 and 6 Hz, while keeping all other algorithm parameters at their default values. Spectral exponents were averaged across all available channels for each individual.

The pre‐processing and analysis of the EEG during visual chirp stimulation was partly based on previously used methodology [34]. Per recording, we re‐referenced the data to the Fz electrode and, per chirp trial, extracted the segment between 2 s before onset (allowing for baseline correction) and 8 s after onset (enabling observation of potential stimulation after‐effects). We averaged the segmented data across electrodes O1, O2, and Pz to approximate a centred occipital electrode location and to reduce noise. We computed each trial's time‐frequency components (5–125 Hz) using Morlet wavelets with 1‐Hz resolution, logarithmically increasing from 3 to 10 cycles in width. We averaged the resultant time‐frequency representations across the 12 chirp trials. Next, we defined the chirp response as the decibel (dB) power change in the EEG response relative to the 1.6–0.1 s pre‐stimulus window. We included the pre‐stimulus window of the participant's first eligible recording day as the baseline for the remaining days, as we anticipated daily variations in 1/f activity that may lead to under‐ or overestimation of effects when using dB‐conversion [42]. We subsequently separated the chirp response into a ‘driving’ and ‘harmonic’ response, averaging the power change across −1 to 1 Hz bins surrounding the stimulation frequency (driving response) and doubles of the stimulation frequency (harmonic response) (Figure 1). We further decomposed the driving and harmonic responses into responses at a low (10–18 Hz), medium (19–26 Hz), and high (27–40 Hz) stimulation frequency range, based on previous work [33, 34]. We performed a *post hoc* analysis (Supporting Information S1) to determine whether visual chirp‐related changes could be attributed to changes in background activity, response changes, or both, given that we used the first day's pre‐stimulus window as the baseline for the remaining days.

### 2.4 Approximation of ASM Load

To approximate daily ASM loads, we modelled blood medication levels over time [43]. The model received as its input the intake of ASMs at the standard morning and afternoon instances of 8 a.m. and 6 p.m. ASM ingestions with other time stamps, as recorded in the patient medical records, were added to the nearest instance. We normalised the ASM doses to the defined daily dose [44]. We estimated hourly ASM load beginning from 3 weeks pre‐admission, assuring steady‐state before tapering, until discharge by first‐order pharmacokinetic modelling [45]. ASM half‐lives were obtained from the literature [2]. We defined the total ASM load relative to the at‐home medication load, ML, for each participant and day *d* by:

$$\mathrm{ML}\left(d\right)=\frac{\sum_{m=1}^{n}{\mathrm{BC}}_{m}\left(d\right)}{\sum_{m=1}^{n}{\mathrm{BC}}_{m}\left(d_{0}\right)}$$

with BC denoting the estimated blood level of ASM type m (out of *n* total types of ASM) indexed at 2 p.m., and d_0_ indicating the last day of unaltered intake. Finally, we converted the ASM load into a daily index of ASM load reduction MLR by:

$$\mathrm{MLR}\left(d\right)=1-\mathrm{ML}\left(d\right)$$

**FIGURE 2:**
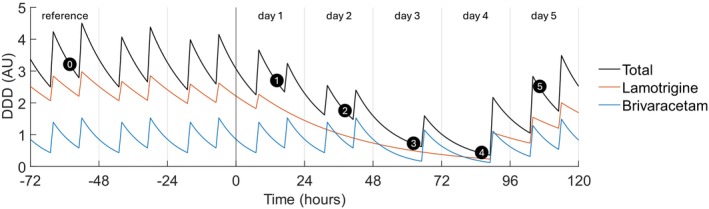
Simulation of anti‐seizure medication (ASM) load for an example participant. Per ASM type, doses were normalised to the defined daily dose (DDD) and their elimination was modelled by first‐order kinetics, rendering hourly estimates of ASM load. The ASM load at 2 p.m. of each admission day, summed across medication types, was expressed relative to the summed value at 2 p.m. of the last day with unchanged home medication (the third day before admission in this example, labelled as ‘reference’). We associated the reduction of the normalised, summed ASM load with the electroencephalographic (EEG) markers. In the plot, (0) indicates 2 p.m. on the reference day, (1) to (4) indicate the 2 p.m. measurement time points at the first four admission days, and (5) indicates the 10 a.m. measurement point on day 5, when the measurement was advanced to the morning to anticipate discharge of the participant around noon. Time is given in hours relative to midnight on the first day of admission. AU, arbitrary units.

Figure 2 exemplifies the simulation of the ASM load.

### 2.5 Data Selection

We anticipated that sleep [46] and seizures [47, 48, 49] could independently drive excitability alterations and thereby potentially obscure ASM withdrawal effects. The correlation between seizure risk and ASM levels could additionally cause multicollinearity in multivariate regression analyses, which may compromise the reliability of regression effect estimates [50]. We, therefore, excluded recordings marked as sleep fragments and recordings obtained less than 4 h after focal impaired awareness seizures or less than 12 h after focal to bilateral tonic–clonic seizures (fbTCS). Sleep scoring was done by two authors (SRG and ARvNA), who each reviewed half of the acquired EEG recordings in 30‐s epochs according to American Academy of Sleep Medicine guidelines [51]. Cases of doubt were presented to the second reviewer and discussed with GHV in case of disagreement. We labelled all recordings that contained at least one epoch of sleep as a sleep fragment.

Resting state EEG recordings were included only if they contained at least 120 s of artifact‐free data. EEG recordings during visual chirp stimulation were included only if they contained the responses to 12 consecutive stimulation trials.

### 2.6 Statistical Analysis

We used custom‐written code in MATLAB R2023b (The MathWorks Inc., Natick, MA, USA) to perform linear mixed‐effects regression analyses, examining the impact of multiple factors on the EEG markers. We included random intercepts to account for participant‐dependent EEG marker baselines. We first explored the effects of sleep and prior seizures on the EEG markers using univariate analysis. Next, we assessed the impact of ASM load reduction on the EEG markers using univariate analyses. Additionally, we examined whether pNSD influenced the EEG markers (*p* < 0.10) in univariate analyses. If so, we proceeded to test the effects of ASM load reduction in multivariate analyses while accounting for pNSD. Each regression analysis modelled the effect on a specific EEG marker: the spectral exponent of resting state EEG, the average driving visual chirp response, or the average harmonic visual chirp response. If either of the two average visual chirp responses was significantly predicted by ASM load reduction, we separately evaluated the low, medium, and high stimulation frequency bands. We applied Holm‐Bonferroni adjustment of *p*‐values in case of multiple comparisons exclusively for univariate analyses. We considered estimated effects with *p*‐values < 0.05 as statistically significant.

## 3 Results

### 3.1 Study Population

We included 48 participants with suspected refractory focal epilepsy between June 2022 and October 2023 (Table 1). The median admission length was 4 days (range 2–5) and the total number of admission days amounted to 228, during which 257 seizures were recorded in 36 (75%) participants. Presurgical evaluation revealed generalised epilepsy in one participant. This participant experienced a generalised myoclonic‐tonic–clonic seizure, which we for pragmatic reasons labelled as an fbTCS in the analyses.

**TABLE 1:** Participant demographic and clinical characteristics.

Participant variable	Value
Age, median (range)	34 (16–62) years
Females, *n* (%)	19 (40%)
Monthly seizure frequency, *n* (IQR)	3 (1–12)
Years since first seizure, *n* (IQR)	10 (6–22)
MRI positive, *n* (%)	37 (77%)
Seizure types*, *n* (%)	
Focal impaired awareness seizure	35 (70%)
Focal to bilateral tonic–clonic seizure	34 (68%)
Focal aware seizure	17 (35%)
Seizure onset region, *n* (%)	
Temporal	29 (60%)
Unknown	8 (17%)
Frontal	6 (13%)
Parietal	3 (6%)
Central	1 (2%)
Occipital	1 (2%)
Concomitant ASMs, *n* (%)	
Two ASMs	25 (52%)
One ASM	17 (35%)
Three ASMs	5 (10%)
Four ASMs	1 (2%)
ASM tapering during admission, *n* (%)	48 (100%)
ASMs reduced during admission*, *n* (%)	
Lacosamide	18 (38%)
Other	13 (27%)
Levetiracetam	11 (23%)
Lamotrigine	11 (23%)
Carbamazepine	9 (19%)
Brivaracetam	8 (17%)
Recorded seizures during admission, *n*	
Focal impaired awareness seizure	98
Focal aware seizure	70
Focal unspecified awareness seizure	57
Focal to bilateral tonic–clonic seizure	25
Generalised myoclonic seizure	6
Generalised myoclonic‐tonic–clonic seizure	1

*Note:* Asterisks (*) indicate the possibility of multiple occurrences per participant.

Abbreviations: ASM, anti‐seizure medication; IQR, interquartile range.

### 3.2 EEG Recordings

**FIGURE 3:**
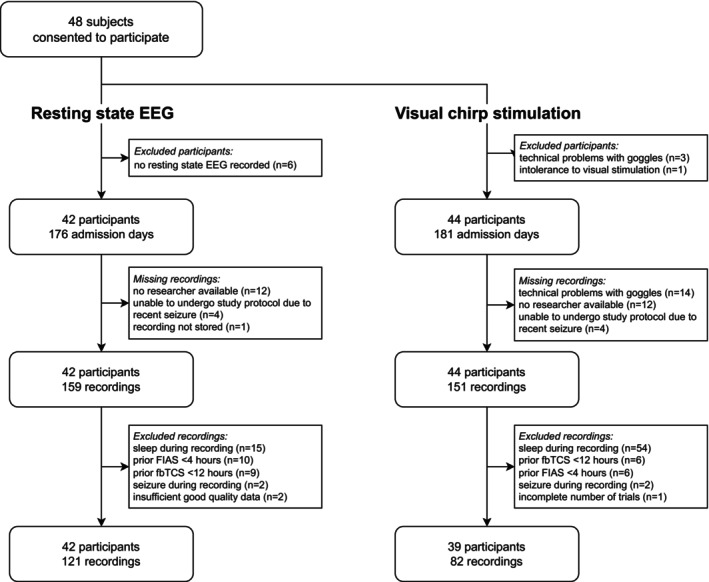
Flowchart illustrating the study participant and electroencephalography (EEG) recording selection procedure. fbTCS, focal to bilateral tonic–clonic seizure; FIAS, focal impaired awareness seizure.

We obtained 159 resting state EEG recordings in 42 participants and 151 EEG recordings during visual chirp stimulation in 44 participants (Figure 3). Selection of recordings eligible for analysis resulted in 121 resting state EEG recordings, with a median of three recordings per participant (range 1–5), and 82 visual chirp stimulation recordings, with a median of three recordings per participant (range 1–5). We rejected a median of 10 artifactual channels (range 1 to 16) from the resting state EEG recordings of each participant prior to spectral analysis.

### 3.3 Effect of ASM Load Reduction

The spectral exponent of resting state EEG and the visual chirp response were impacted by recent focal impaired awareness seizures and fbTCS before the recording, as well as by sleep during the recording (Table S1), justifying the exclusion of these respective recordings from further analysis of ASM load reduction effects. A full overview of the regression analysis results is provided in Table S1 and a summary in Table 2.

**TABLE 2:** Summary of the effects of anti‐seizure medication (ASM) tapering, prior seizures, and sleep on the electroencephalography (EEG) markers.

	ASM tapering^a^	Prior pNSD^b^	Prior fbTCS < 12 h^b^	Prior FIAS < 4 h^b^	Sleep during recording^b^
Spectral exponent of resting state EEG	↓	=	=	↑	↑
Chirp driving response	=	=	=	↑	↓
Chirp harmonic response	↑	↑	=	↑	=
Low stimulation frequencies	=	N.A.	N.A.	N.A.	N.A.
Medium stimulation frequencies	=	N.A.	N.A.	N.A.	N.A.
High stimulation frequencies	↑	N.A.	N.A.	N.A.	N.A.

*Note:* Significant (*p* < 0.05) positive effects are represented by upward arrows, significant negative effects are represented by downward arrows, and absence of effects is indicated by equal signs.

Abbreviations: fbTCS, focal to bilateral tonic–clonic seizure; FIAS, focal impaired awareness seizure; N.A., not applicable.

^a^ Effects estimated in a multivariate regression analysis that corrected for partial nocturnal sleep deprivation (pNSD) before the measurement.

^b^ Effects estimated in a univariate regression analysis.

#### 3.3.1 Resting State EEG

**FIGURE 4:**
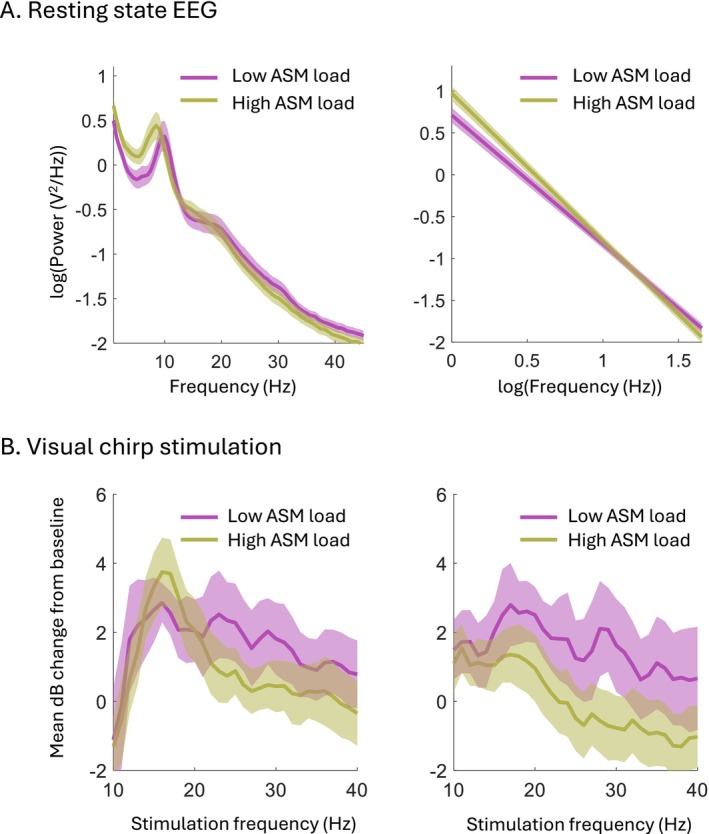
Illustration of effects of anti‐seizure medication (ASM) load reduction on the spectral exponent of resting state electroencephalography (EEG) and the visual chirp response. Results are shown for low ASM loads (> 75% reduction compared to pre‐tapering values) and high ASM loads (< 25% reduction compared to pre‐tapering values). (A) Average resting state EEG power spectra (left) and aperiodic fits (right), with their 95% confidence intervals. ASM tapering yielded a less negative spectral exponent, which is equivalent to a flatter slope of the aperiodic fit in the log–log space. This result was confirmed (*p* < 0.05) by mixed linear regression analysis. The shift of the exponent mediated by ASM tapering seems primarily driven by a power reduction in the EEG power across the delta‐to‐alpha frequency range. (B) Average EEG responses at driving (left) and harmonic (right) frequencies assessed per chirp stimulation frequency, with their 95% confidence intervals. ASM tapering specifically increased the harmonic response for the high (i.e., 26–40 Hz) stimulation frequencies. This result was confirmed (*p* < 0.05) by linear mixed‐effects regression analysis.

ASM load reduction resulted in a less negative spectral exponent of resting state EEG (estimate [95% CI]: 0.09 [0.02 to 0.17], *p* = 0.02). The spectral exponent was not affected by pNSD (estimate [95% CI]: 0.03 [−0.02 to 0.07], *p* = 0.23). The exponent decrease seemed primarily driven by a reduction of the delta, theta, and alpha power (Figure 4A).

#### 3.3.2 Visual Chirp Stimulation

ASM load reduction did not increase the average driving response (estimate [95% CI]: 0.57 dB [−0.47 to 1.60], *p* = 0.28) but did increase the average harmonic response (estimate [95% CI]: 1.92 dB [0.64 to 3.19], *p* = 0.004) to visual chirp stimulation. This increase was specifically observed in the harmonic response to medium (estimate [95% CI]: 1.88 dB [0.45 to 3.31], *p* = 0.02) and high (estimate [95% CI]: 2.58 dB [0.89 to 4.27], *p* = 0.01) stimulation frequencies (Figure 4B). The average harmonic response was also increased by pNSD (estimate [95% CI]: 0.86 dB [0.08 to 1.64], *p* = 0.03). The effect of ASM load reduction on the average harmonic response persisted when accounting for pNSD effects in multivariate analyses (estimate [95% CI]: 1.62 dB [0.26 to 2.98], *p* = 0.02). Furthermore, multivariate analyses revealed that ASM load reduction specifically increased the response to high stimulation frequencies (estimate [95% CI]: 2.31 dB [0.51 to 4.12], *p* = 0.01). A *post hoc* analysis showed that the effect is likely driven by a combination of actual response changes and background activity changes (Supporting Information S1).

## 4 Discussion

We found a significant correlation between ASM load and putative EEG markers of cortical excitability in people with refractory epilepsy who underwent multi‐day EEG recordings during preclinical screening. Reduction of ASM load yielded a less negative spectral slope of the resting state EEG, primarily due to a decrease in spectral slowing. Prior in silico and in vivo studies that supported its use as a marker of neuronal excitability reported altered slopes due to spectral changes across frequencies up to 75 Hz [15, 17, 18, 20]. Our finding seems less specific for a general increase in brain excitability, as we observed no increase in the higher frequency range. Visual chirp stimulation revealed an enhanced second‐order harmonic response to higher stimulation frequencies during ASM tapering. The augmented chirp response likely resulted from both increased high gamma activity and an increased response to visual perturbations. Although neither the spectral exponent nor the visual chirp response was exclusively indicative of changes in excitability, both markers might be used to measure aspects of the E/I balance during ASM titration.

The spectral exponent of resting state EEG data became less negative with declining ASM load. As a smaller spectral exponent has been linked to a higher E/I ratio [15], this result might suggest an overall increase in neuronal excitability. We, however, observed that a reduction in ASM load primarily led to a decrease in EEG power in the delta‐to‐alpha frequency range, without affecting the higher frequencies. While acute ASM withdrawal has been associated with a broadband decrease in EEG power [52], the largest effects were indeed observed in the lower frequency range [52, 53]. The spectral exponent change may thus reflect a reversal of the spectral slowing reportedly induced by certain ASMs, such as carbamazepine and oxcarbazepine, which was hypothesised to reflect ASM‐related changes in cognitive abilities [54, 55, 56, 57], rather than reflecting changes in the E/I ratio specific to the epileptic brain [18, 20]. The spectral exponent may, therefore, be less suitable for tracking acute ASM effects on cortical excitability as a predictor of their efficacy. Nevertheless, we cannot exclude that ASM tapering reduced EEG synchronisation in lower frequency bands as a sign of reduced inhibition [58].

A reduction in ASM load was associated with a stronger second‐order harmonic EEG response to visual chirp stimulation in the high (27–40 Hz) frequency range. In people with migraine, a partly overlapping chirp range (22–32 Hz) disclosed enhanced pre‐ictal visual responsivity, presumably reflecting increased cortical excitability towards an attack [34]. We are not aware of prior visual chirp stimulation studies in people with epilepsy, yet transcranial magnetic stimulation, as an alternative perturbational modality, revealed increasing cortical excitability during ASM tapering by reduced resting motor thresholds [49]. Notably, our finding that the visual chirp response may decrease after an fbTCS but increases after a focal impaired awareness seizure parallels observations made in the prior transcranial magnetic stimulation study [49]. Although our study was not powered to detect post‐ictal effects, the congruence might further support the notion that the visual chirp response seems able to track cortical excitability.

Visual chirp stimulation revealed increased neuronal activity after ASM withdrawal specifically in the gamma frequency range (54–80 Hz). This may have signaled increased susceptibility to seizures. Enhanced gamma band phase clustering, elicited by transcranial magnetic stimulation, distinguished people with juvenile myoclonic epilepsy from controls [59]. This phenomenon, when induced by visual stimulation, also correlated with impending photoparoxysmal responses in photosensitive epilepsy [27]. Furthermore, children with febrile seizures demonstrated altered gamma‐range steady‐state visual evoked potential spectral components compared to healthy controls [60]. Aside from the notion that perturbing brain activity may reveal increased cortical responsivity indicating increased seizure susceptibility, direct effects of ASM tapering on background gamma activity might have separately led to increased gamma responsivity. Withdrawal of voltage‐gated sodium channel antagonists, such as carbamazepine and lamotrigine, which constitute the majority of tapered ASMs in our study, may explain the enhanced gamma band activity [61]. Tapering agents potentiating the GABAergic system, such as valproate but possibly also levetiracetam [62], could have lowered alpha power [63, 64], which also might have facilitated an increase in gamma power [65, 66, 67]. The role of such ASM effects on background gamma oscillatory activity, however, appears limited given the minimal visual difference in EEG gamma power we observed between low and high ASM loads.

Our study leveraged multi‐day EEG recordings to evaluate the relationship between ASM tapering and EEG markers of cortical excitability, while minimising potential confounding by the circadian rhythm [68], sleep deprivation [69, 70], and rescue medication intake [71, 72]. With a concise and simple experimental protocol, which has the potential for easy clinical implementation, we identified quantitative EEG biomarkers revealing acute ASM effects on cortical excitability. Our study, however, also has a few limitations. First, we were unable to relate the EEG markers to actual ASM efficacy measures, as this would require prolonged monitoring of seizure frequency and a stable ASM intake. We, instead, exploited individually modelled ASM load, which has previously been correlated with cortical excitability [49], a critical driver of seizure susceptibility [73]. Nevertheless, the effects of acute ASM withdrawal on the brain activity contrast with the outpatient situation of slow ASM dose modifications, thus underscoring the need for prospective studies to relate our markers with ASM titration and efficacy. Second, due to the diversity of ASMs, we cannot attribute certain withdrawal effects to specific medication types. Despite the variation in working mechanisms, however, our study already shows a clear correlation between the aggregate ASM load and both the visual chirp response and the spectral exponent. Future studies may disentangle the relative contributions of different ASM types to modulation of comparable EEG markers of cortical excitability. Third, particularly during the visual chirp stimulation that followed the resting state recording, we commonly noted signs of sleep in participants. Randomising the order of the measurements could have reduced sleep risk, but we prioritised keeping the conditions the same for the benefit of day‐to‐day comparisons. We, therefore, classified each EEG recording as sleep or wake and excluded sleep recordings from analysis. Lastly, we aimed to exclude post‐ictal effects on the EEG read‐outs used for ASM effects analysis. Altered spontaneous EEG activity was observed in drug‐naive individuals from a few minutes after focal seizures up to just over half an hour after fbTCS [74]. Cortical responses may differ from baseline for several hours after focal seizures [49] and, in untreated individuals compared to healthy controls, up to 2 days following a first fbTCS [75]. Some common ASMs have been found to reduce post‐ictal symptoms or duration [76], which may have suppressed post‐ictal effects in our cohort. Omitting measurements within 4 h after a focal impaired awareness seizure or 12 h after an fbTCS may, thus, not have excluded all post‐ictal effects, but presumably rendered their impact negligible.

The potential of the spectral exponent of resting state EEG and the visual chirp response as accessible EEG biomarkers of ASM efficacy should be confirmed in future studies involving long‐term participant follow‐up with seizure diaries and evaluations during stable ASM doses. Eventually, the implementation of the markers would need normative values to reduce the delay to individually optimised treatment regimens.

## Author Contributions

**Silvano R. Gefferie, Else A. Tolner, Arn M. J. M. van den Maagdenberg, Roland D. Thijs:** conceptualization. **Silvano R. Gefferie, Arthur R. van Nieuw Amerongen:** data curation. **Silvano R. Gefferie, Arthur R. van Nieuw Amerongen:** formal Analysis. **Arn M. J. M. van den Maagdenberg, Roland D. Thijs:** funding acquisition. **Silvano R. Gefferie, Arthur R. van Nieuw Amerongen:** investigation. **Silvano R. Gefferie, Arthur R. van Nieuw Amerongen, Gerhard H. Visser, Mark van de Ruit, Roland D. Thijs:** methodology. **Else A. Tolner, Mark van de Ruit, Arn M. J. M. van den Maagdenberg, Roland D. Thijs:** supervision. **Silvano R. Gefferie, Arthur R. van Nieuw Amerongen:** visualisation. **Silvano R. Gefferie, Arthur R. van Nieuw Amerongen:** writing – original draft. **Silvano R. Gefferie, Arthur R. van Nieuw Amerongen, Gerhard H. Visser, Maeike Zijlmans, Else A. Tolner, Mark van de Ruit, Arn M. J. M. van den Maagdenberg, Roland D. Thijs:** writing – review and editing.

## Conflicts of Interest

R.D.T. reports lecture and consultancy fees from Angelini, Eisai, LivAssured, UCB, Theravarance, Zogenix, Novartis, and Arvelle, and grants from EpilepsieNL, Michael J. Fox Foundation, and NewLife Wearables.

## Supporting information


Data S1.

## Data Availability

The data that support the findings of this study are available from the corresponding author upon reasonable request.
