# Supplementary material for: EEG Spectral Exponents and Visual Chirp Responses Mirror Anti‐Seizure Medication Load in Refractory Focal Epilepsy
Source: Ann Clin Transl Neurol. 2025 Apr 21;12(6):1265–75. doi: 10.1002/acn3.70045 (PMC12172104; doi:10.1002/acn3.70045)
Supplement: Supplementary file 1 — Data S1. [file ACN3-12-1265-s001.zip › acn370045-sup-0001-ACTN_suppl. table 1_rebuttal_1_clean_[date 2025-02-26].docx]

| **Supplementary Table 1.** Full overview of linear mixed-effects regression results. | | | | |
| --- | --- | --- | --- | --- |
| **Predictor variable** | **Response variable** | **Estimate (95% CI)** | **Sign.** | **df** |
| *Univariate regression* | | | | |
| ASM tapering | Spectral exponent of resting state EEG | 0.09 (0.02 to 0.17) | 0.02* | 119 |
|  | Chirp driving response | 0.57 (-0.47 to 1.60) | 0.28 | 80 |
|  | Chirp harmonic response | 1.92 (0.64 to 3.19) | 0.004* | 80 |
|  | Low stimulation frequencies (10-18 Hz) | 0.86 (-0.12 to 1.85) | 0.085 | 80 |
|  | Medium stimulation frequencies (19-26 Hz) | 1.88 (0.45 to 3.31) | 0.021* | 80 |
|  | High stimulation frequencies (27-40 Hz) | 2.58 (0.89 to 4.27) | 0.01* | 80 |
| Prior pNSD | Spectral exponent of resting state EEG | 0.03 (-0.02 to 0.07) | 0.23 | 119 |
|  | Chirp driving response | 0.20 (-0.39 to 0.79) | 0.51 | 119 |
|  | Chirp harmonic response | 0.86 (0.08 to 1.64) | 0.03* | 119 |
| Prior fbTCS (<12 hours) | Spectral exponent of resting state EEG | -0.07 (-0.16 to 0.01) | 0.10 | 138 |
|  | Chirp driving response | -0.90 (-2.0 to 0.21) | 0.11 | 92 |
|  | Chirp harmonic response | -0.78 (-2.35 to 0.79) | 0.33 | 92 |
| Prior FIAS (<4 hours) | Spectral exponent of resting state EEG | -0.10 (-0.19 to 0.00) | 0.049* | 138 |
|  | Chirp driving response | 1.29 (0.28 to 2.31) | 0.01* | 92 |
|  | Chirp harmonic response | 1.49 (0.03 to 2.95) | 0.046* | 92 |
| Sleep during recording | Spectral exponent of resting state EEG | -0.41 (-0.77 to -0.04 ) | 0.03* | 153 |
|  | Chirp driving response | -0.59 (-1.11 to -0.07) | 0.03* | 146 |
|  | Chirp harmonic response | -0.65 (-1.36 to 0.07) | 0.08 | 146 |
| *Multivariate regression* | | | | |
| ASM tapering  Prior pNSD | Chirp harmonic response | 1.62 (0.26 to 2.98)  0.53 (-0.29 to 1.35) | 0.02*  0.20 | 79 |
| ASM tapering  Prior pNSD | Low stimulation frequencies | 0.65 (-0.41 to 1.72)  0.32 (-0.27 to 0.91) | 0.23  0.28 | 79 |
| ASM tapering  Prior pNSD | Medium stimulation frequencies | 1.45 (-0.07 to 2.97)  0.69 (-0.19 to 1.58) | 0.06  0.12 | 79 |
| ASM tapering  Prior pNSD | High stimulation frequencies | 2.31 (0.51 to 4.12)  0.51 (-0.63 to 1.64) | 0.01*  0.38 | 79 |

Asterisks (*) denote statistical significance at *p* < 0.05. When the visual chirp response was evaluated across separate stimulation frequency bins using univariate regression, we applied a Holm-Bonferroni correction to the *p*-values of the estimated effects. ASM: anti-seizure medication; CI: confidence interval; df: degrees of freedom; EEG: electroencephalography; fbTCS: focal to bilateral tonic-clonic seizure; FIAS: focal impaired awareness seizure; pNSD: partial nocturnal sleep deprivation; sign.: significance.
