# Supplementary material for: EEG Spectral Exponents and Visual Chirp Responses Mirror Anti‐Seizure Medication Load in Refractory Focal Epilepsy
Source: Ann Clin Transl Neurol. 2025 Apr 21;12(6):1265–75. doi: 10.1002/acn3.70045 (PMC12172104; doi:10.1002/acn3.70045)
Supplement: Supplementary file 1 — Data S1. [file ACN3-12-1265-s001.zip › acn370045-sup-0002-ACTN_suppl. text_rebuttal_1_[date 2025-02-26].docx]

# **Supplementary Text**

*EEG spectral exponents and visual chirp responses mirror anti-seizure medication load in refractory focal epilepsy*

Silvano R. Gefferie MSc.^1,2^*, Arthur R. van Nieuw Amerongen MSc.^1,2^*, Gerhard H. Visser MD PhD^1^, Maeike Zijlmans MD PhD^1,3^, Else A. Tolner PhD^2,4^, Mark van de Ruit PhD^2,5^, Arn M.J.M. van den Maagdenberg PhD^2,4^, and Roland D. Thijs MD PhD FRCP FEAN^1,2,6^

^1^Stichting Epilepsie Instellingen Nederland (SEIN), Heemstede, Achterweg 3, 2103 SW, The Netherlands

^2^Department of Neurology, Leiden University Medical Centre, Albinusdreef 2, 2333 ZA, Leiden, The Netherlands

^3^Department of Neurology, University Medical Center Utrecht, Heidelberglaan 100, 3584 CX, Utrecht, The Netherlands

^4^Department of Human Genetics, Leiden University Medical Centre, Albinusdreef 2, 2333 ZA, Leiden, The Netherlands

^5^Department of Biomechanical Engineering, Delft University of Technology, Mekelweg 2, 2628 CD Delft, The Netherlands

^6^Department of Clinical & Experimental Epilepsy, UCL Queen Square Institute of Neurology, London WC1N 3BG, United Kingdom

*These authors contributed equally to this work

***Post hoc* analysis to assess the factors contributing to the changes found in the visual chirp response**

In our study, we expressed the EEG response to visual chirp stimulation as the decibel change from a pre-stimulus window, similar to earlier visual chirp stimulation studies^1,2^:

$$\text{dB change from baseline}=10 \cdot\log_{10} (\frac{\text{response EEG activity}}{\text{pre-stimulus EEG activity}})$$

A recent study demonstrated that a valid comparison of responses derived by such a baseline correction requires the 1/f-component of the EEG spectrum to be constant across measurements.^3^ This is especially relevant in our study where we obtained longitudinal measurements, rather than cross-sectional observations as in the previous clinical study with visual chirp stimulation^2^. Since a day-to-day variation of pre-stimulus activity is likely (varying signal quality, stress levels, et cetera), we used the pre-stimulus window of the first recording day as the baseline activity for the rest of the patient’s measurements.

The linear mixed model we used specifically relates visual chirp responses to anti-seizure medication (ASM) load. Stochastic day-to-day variation in the pre-stimulus window, unrelated to ASM load, is not expected to affect the model outcomes. However, if pre-stimulus activity does depend on ASM load, it could deflate or inflate the relationship between visual chirp response and ASM load. An observed response change may then be a combination of a true response change and a pre-stimulus activity change. A *post hoc* evaluation could help to determine if there is ASM load-dependent pre-stimulus activity variation and its impact on the model results.

We reported an increased EEG harmonic response to high frequency flash stimulation (27 to 40 Hz) after ASM tapering (estimate (95% CI): 2.58 dB (0.89 to 4.27), *p* = 0.01). For the *post-hoc* analysis, we explored whether this increase may be (partly) driven by an ASM load-dependent change in pre-stimulus activity, using the same linear mixed model statistics as in the main analysis. Specifically, we tested the relationship between ASM load and the pre-stimulus EEG band power corresponding to the second-order harmonics of the high chirp stimulation frequencies band (i.e. 54 to 80 Hz). This yielded a positive relationship (*p* = 0.06), which, while non-significant, does indicate that pre-stimulus activity variation might have contributed to the main chirp response findings. We explored how a same-day baseline-corrected EEG response would correlate to ASM load. We found a positive but non-significant correlation (estimate (95% CI): 0.32 dB (-0.44 to 1.09), *p* = 0.40). This result should be interpreted with caution as the requirement of equal 1/f activity in the pre-stimulus window was violated. Taken together, these results suggest that the observed increased EEG harmonic visual chirp response after ASM load reduction should be considered as a combination of ASM load-dependent variation in both pre-stimulus activity and chirp response activity.

We also examined the dynamics of EEG power in the 54-to-80 Hz band (i.e., harmonics of high chirp stimulation frequencies) for pre-stimulus activity and response window activity, without any baseline correction (Figure 1). We pooled all measurements at high medication load (≤25% tapered) and low medication load (≥75% tapered). Median EEG power was higher in the low medication group for both the pre-stimulus activity and the response activity (median difference 0.03 μV^2^), but the difference was greater for response activity (median difference 0.06 μV^2^). Also, the effect size for the response activity (Cohen’s *d* 0.17) was more than double the effect size for the pre-stimulus activity (Cohen’s *d* 0.08). This suggests that, although pre-stimulus activity contributed to the observed effect, the response activity appears to be the main driver. It may be recognized that these effects might appear small, but this comparison of a high and low medication group does not take into account the individual trends as the linear mixed model does.

**Figure 1** – EEG power in the high harmonic frequencies band (54 to 80 Hz) during high medication levels (<25% tapered) and low medication levels (>75% tapered). **Left**: EEG power during average *pre-stimulus* *window*. **Right:** EEG power during average *response to 27to40 Hz stimulation*. Horizontal lines indicate median value. Of note, this representation does not leverage the fact that we did serial measurements in individuals, which was addressed by the linear mixed model statistics instead.

**References**

1.	Gantenbein AR, Sandor PS, Goadsby PJ, Kaube H. Chirp stimulation: H-response short and dynamic. Cephalalgia 2014;34(7):554–558.

2.	Perenboom MJ, van de Ruit M, Zielman R, et al. Enhanced pre-ictal cortical responsivity in migraine patients assessed by visual chirp stimulation. Cephalalgia 2020;40(9):913–923.

3.	Gyurkovics M, Clements GM, Low KA, et al. The impact of 1/f activity and baseline correction on the results and interpretation of time-frequency analyses of EEG/MEG data: A cautionary tale. Neuroimage 2021;237:118192.
